# Sleep duration, daytime napping, and risk of incident stroke: Nuances by metabolic syndrome from the China health and retirement longitudinal study

**DOI:** 10.3389/fcvm.2022.976537

**Published:** 2022-09-02

**Authors:** Yuanyuan Fang, Yuqin He, Yanzhu Huang, Lusen Ran, Wenhui Song, Jiahuan Hao, Di Yao, Rong Li, Dengji Pan, Tingting Qin, Minghuan Wang

**Affiliations:** ^1^Department of Neurology, Tongji Hospital, Tongji Medical College, Huazhong University of Science and Technology, Wuhan, China; ^2^Department of Biliary-Pancreatic Surgery, Tongji Hospital, Tongji Medical College, Huazhong University of Science and Technology, Wuhan, China

**Keywords:** nighttime sleep duration, total sleep duration, daytime napping, incident stroke, metabolic syndrome

## Abstract

**Background and purpose:**

The relationship between sleep duration and stroke are inconclusive in China, especially in those individuals with metabolic syndrome. We aimed to investigate the association between sleep duration and incident stroke in participants with metabolic syndrome or its specific components in China.

**Materials and methods:**

Data were taken from the 2011 and 2015 waves of China Health and Retirement Longitudinal Study (CHARLS). Habitual sleep duration (≤6, 6∼8 [reference], >8 h), daytime napping (0, 1∼60 [reference], and >60 min) were determined by self-reported questionnaires. Metabolic syndrome was defined by blood assessment and biomarkers combined with self-reported doctors’ diagnosis. Incident stroke was determined by reported stroke from 2011 to 2015 wave. Cross-sectional and longitudinal associations between sleep and (incident) stroke at baseline and 4-year follow-up period were tested among the population with metabolic syndrome and its components.

**Results:**

A U-shaped relationship was observed between sleep duration and stroke in cross-sectional analysis. Sleep ≤ 6 h/night had a greater risk of incident stroke (hazard ratio [HR] 1.65; 95% confidence interval [CI] 1.04–2.61) compared with sleep 6∼8 h/night. And the HR of stroke was 1.62 (95%CI, 1.03–2.53) for sleep < 7 h/day compared to 7∼9 h/day. These associations were more evident in the female and individuals aged 45–65 years. Furthermore, the effect of short sleep duration on incident stroke was different in each component of metabolic syndrome, which was more pronounced in participants with elevated blood pressure. And a significant joint effect of sleeping ≤ 6 h/night and no napping on risk of stroke was observed (HR 1.82, 95%CI 1.06–3.12).

**Conclusion:**

Short sleep duration was an independent risk factor for incident stroke, especially among females, individuals aged 45–65 years, or those with some components of metabolic syndrome, such as hypertension. Napping could buffer the risk of short sleep duration on incident stroke.

## Introduction

Stroke is a major public health concern, representing the leading cause of death and disability-adjusted life-years (DALYs) at the national level in China (1). The Global Burden of Disease Study 2019 reported that there were 3.94 million new stroke cases, 2.19 million deaths and 45.9 million DALYs due to stroke in China (2). The stroke incidence is multifactorial and traditional risk factors cannot explain the entire stroke risk (2, 3). A growing evidence suggests sleep patterns, especially nighttime sleep duration and daytime napping could be specific risk factors of stroke (4, 5).

Sleep is basic need for human beings. According to the National Sleep Foundation’s recommendations, the optimal night sleep duration for adults is 7–8 h (6). Certainly, adequate sleep duration is essential for maintaining physical and psychological health. Sleep too much or too little is associated with chronic health conditions such as stroke, obesity, hypertension, diabetes, and dyslipidemia (5, 7–10). Some cross-sectional and prospective studies have investigated the association between sleep duration and stroke (11–13). What’s more, various studies indicated a J-shaped (14–16), U-shaped (17) or non-linearity (18, 19) relationships between sleep duration and stroke. These conflicting results might be explained by the differences in cohort characteristics, sample size and adjustment of confounders.

As a supplement to nighttime sleep, napping is considered as a healthy habit (20) and about 58% Chinese has the habit of napping (21). Conversely, previous study identified that extended napping can increase the risk of stroke (22). However, other study indicated that napping can compensate for chronic sleep loss (23). The exact association between the total sleep duration (with and without a nap) and incident stroke, and whether napping could affect the association of nighttime sleep duration on stroke has not been understood.

As we know, metabolic syndrome (Mets) is a global public health concern, which is major risk factors for stroke (24, 25), affecting about one-third of the Chinese (26). Recently, a number of studies have indicated excessive or insufficient sleep, and prolonged napping could lead to Mets (27–30). However, the association of sleep duration and stroke among participants with Mets is still less known. To date, few studies have explored the relationship between sleep duration and stroke in different status of hypertension (31).

Based on the above uncertainty, we aimed to investigate the relationship between sleep duration, napping and the risk of stroke in a prospective cohort study of 14,532 middle-aged and elderly Chinese adults. Besides, we would explore the joint effect of nighttime sleep duration and daytime napping on incident stroke. In addition, the relationship between sleep duration and incident stroke among participants with different status of metabolic syndrome and its components would be investigated.

## Materials and methods

### Study population

**FIGURE 1:**
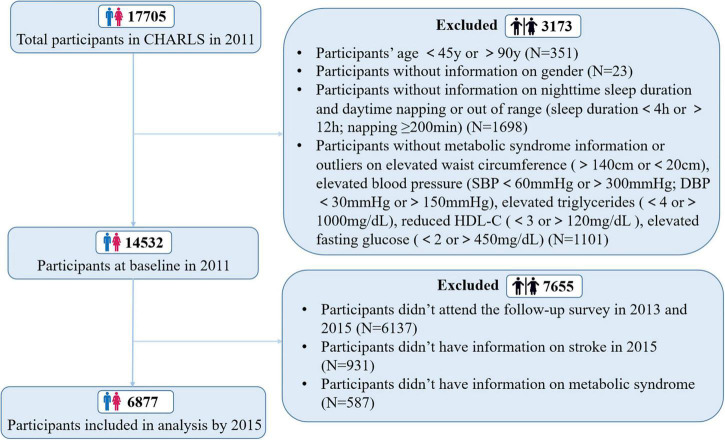
Flow of participants through study.

China Health and Retirement Longitudinal Study (CHARLS) is a nationally representative population-based, prospective survey that enrolled participants aged 45–90 years from 2011. The CHARLS cohort collected information about demographic characteristics, lifestyle habits, and general health status at the individual, family, and community levels every 2 years. For this study, we selected an initial sample of 14,532 respondents at baseline in 2011 that had completed sleep questionnaire, stroke questionnaire, and the biomarkers and blood assessment to investigate the cross-sectional relationship between sleep and stroke. Then eligible participants who completed sleep and stroke questionnaire in the follow-up survey in 2015 were included in the prospective analysis and finally, 6,877 participants included in the longitudinal analysis. More details of inclusion process of study sample can be seen in Figure 1.

### Assessment of sleep duration and daytime napping

Sleep duration and napping were determined by self-reported questionnaires. Nighttime sleep duration was assessed by the following question: “During the past month, how many hours of actual sleep did you get every night? (mean hours per night)” and stratified into three groups: short (≤6 h), normal (6∼8 h) and long (>8 h), as described in another study based on CHARLS (32). Daytime napping was assessed by asking “During the past month, how long did you take naps after lunch?,” and stratified to no napping (0 min), 1∼60 min, and > 60 min napping (32). Additionally, we calculated total sleep duration in 24 h by summing up nighttime sleep duration and daytime napping, as in accordance with a previous study from CHARLS (33). The National Sleep Foundation’s recommends that the normal sleep duration per day is 7–9 h for those aged 45–65 years (6), so total sleep duration was recorded into three levels: short (<7 h), normal (7∼9 h) and long (>9 h).

### Assessment of metabolic syndrome

The presence of Mets was defined by the coexistence of three or more of the following criteria (34, 35): (1) elevated waist circumference: waist circumference ≥ 90 cm in men or ≥ 80 cm in women; (2) elevated triglycerides: triglycerides ≥ 150 mg/dL or a self-reported doctors’ diagnosis of dyslipidemia; (3) reduced high-density lipoprotein cholesterol (HDL-C): HDL cholesterol < 40 mg/dL in men, or < 50 mg/dL in women, or self-reported doctors’ diagnosis of dyslipidemia; (4) elevated blood pressure: mean blood pressure ≥ 130/85 mmHg or self-reported hypertension; (5) elevated fasting glucose: fasting glucose ≥ 100 mg/dL or a self-reported doctor’s diagnosis of diabetes.

### Assessment of stroke

Self-reported stroke was assessed by posing the following questions: “Have you been diagnosed with stroke by a doctor”; “When was the condition first diagnosed or known by yourself?”; “Since the last visit/In the last 2 years, has a doctor told you that you had another stroke?”; “When was your most recent stroke?,” an affirmative response was considered as having a stroke (33). The outcome of interest was incident stroke that first occurred after baseline survey but before October 11, 2017.

### Assessment of covariates

The covariates data were selected based on findings from previous studies (5, 13). Information on sociodemographic characteristics, lifestyle behaviors, and clinical or biochemical measures were collected at baseline using semi-structured questionnaires and clinical/biochemical measures. The sociodemographic characteristics included age, gender (male/female), education level (elementary school or below/from middle school to secondary school/Bachelor’s degree or beyond), marital status (married/unmarried), and area of residence (rural/urban). Lifestyle behaviors included smoking status (never/quit/current), drinking status (never/quit/current), and physical activity (walking/moderate/vigorous). Clinical/biochemical measures included the self-report diagnosis of stroke, dyslipidemia, hypertension, diabetes or high blood sugar, body mass index (BMI), waist circumference, triglycerides, HDL-C, systolic and diastolic blood pressure, fasting plasma glucose and glycosylated hemoglobin.

### Statistical analyses

Continuous variables are presented as mean (SD) and compared using variance or Mann-Whitney U tests, whereas categorical variables are presented as count and proportions and compared with chi-square tests. Multivariate logistic regression and cox proportional hazards analysis were used to access the cross-sectional and longitudinal relationship of sleep parameters (nighttime sleep duration, daytime napping, and total sleep duration) and risk of stroke, respectively. Based on previous studies, we used nighttime sleep duration of 6∼8 h/night, daytime napping of 1∼60 min, and total sleep duration of 7∼9 h as the reference group intervals. The potential non-linear trends of sleep duration and incident stroke risk were evaluated by restricted cubic spline regression. We further accessed the joint effect of nighttime sleep duration and daytime napping on the risk of incident stroke, taking nighttime sleep duration of 6∼8 h per night and no napping as the reference groups. Stratified analyses were performed by sociodemographic characteristics (age and gender) and five components of Mets. Furthermore, we performed a sensitivity analysis according to objectively assessed Mets to explore the risk of stroke. Two-sided *P*-value < 0.05 were considered statistically significant. All statistical analyses were conducted using SAS version 9.3 (SAS Institute Inc., Cary, NC, United States).

## Results

### Participants characteristics

A total of 14,532 participants (7,052 men, mean age 59.8 ± 9.5 years) were included in this study (Figure 1). Sample characteristics of these participants by sleep duration categories are presented in Table 1. Overall, 46.0% had short nighttime sleep duration (<6 h/night), while 9.1% had long sleep duration (>8 h/night). Over half of the population (54.2%) took a nap during daytime. Among the individuals with total sleep duration, there were 5121 (35.2%) people who slept less than 7 h per day and 1,782 (12.3%) people who slept more than 9 h per day. Compared with participants reporting normal sleep duration per night/day or 1∼60 min daytime napping, those reporting short or long sleep duration per night/day or daytime napping > 60 min were more likely to be older, females, less educated, unmarried, rural residents, with poor health state and disabilities, and were less likely to be obesity (all *P* < 0.05). In addition, individuals napping > 60 min were more likely to be current smokers and have insufficient physical activity, obesity, hypertension, hyperglycemia, hyperlipidemia and Mets compared to the reference group (Table 1).

**TABLE 1:** Baseline sample characteristics according to sleep categories.

Variable	Total	Nighttime sleep duration				Daytime napping				Total sleep duration			
		≤6 h	6 to ≤8 h	>8 h	*P-*value	0	1 to ≤60 min	>60 min	*P*-value	<7 h	7 to ≤9 h	>9 h	*P*-value
	*n* = 14,532	*n* = 6,685	*n* = 6,528	*n* = 1,319		*n* = 6,656	*n* = 5,342	*n* = 2,508		*n* = 5,121	*n* = 7,629	*n* = 1,782	
**Demographic characteristics**													
Age, years, mean (SD)	59.83 (9.49)	60.43 (9.39)	58.99 (9.31)	60.92 (10.43)	< 0.0001	59.25 (9.18)	60.00 (9.60)	60.98 (9.88)	< 0.0001	60.32 (9.31)	59.23 (9.36)	60.97 (10.31)	< 0.0001
Male, *n* (%)	7,052 (48.53)	3,158 (47.24)	3,265 (50.02)	629 (47.69)	0.005	2,790 (41.92)	2,801 (52.43)	1,448 (57.74)	< 0.0001	2,217 (43.29)	3,893 (51.03)	942 (52.86)	< 0.0001
Education level (missing = 4)					< 0.0001				<0.0001				< 0.0001
Elementary school or below, *n* (%)	6,264 (43.1)	2,992 (44.76)	2,592 (39.72)	680 (51.55)		3,150 (47.34)	2,073 (38.82)	1,029 (41.03)		2,445 (47.74)	3,001 (39.36)	818 (45.90)	
From Middle school to Bachelor’s degree, *n* (%)	7,878 (54.21)	3,536 (52.90)	3,717 (56.97)	625 (47.38)		3,399 (51.08)	3,032 (56.78)	1,434 (57.18)		2,572 (50.22)	4,363 (57.22)	943 (52.92)	
Bachelor’s degree or beyond, *n* (%)	386 (2.66)	156 (2.33)	216 (3.31)	14 (1.06)		105 (1.58)	235 (4.40)	45 (1.79)		104 (2.03)	2,61 (3.42)	21 (1.18)	
Married, *n* (%)	12,859 (88.49)	5,859 (87.64)	5,867 (89.87)	1,133 (85.90)	< 0.0001	5,840 (87.74)	4,769 (89.27)	2,227 (88.80)	0.0284	4,462 (87.13)	6,834 (89.58)	1,563 (87.71)	0.0001
Rural, *n* (%)	12,886 (88.67)	5,930 (88.72)	5,729 (87.77)	1,227 (93.10)	< 0.0001	5,937 (89.22)	4,648 (87.02)	2,279 (90.87)	< 0.0001	4,548 (88.83)	6,696 (87.78)	1,642 (92.20)	< 0.0001
**Lifestyle factors**													
Smoking status (missing = 5)					0.3257				< 0.0001				<0.0001
Never, *n* (%)	8,767 (60.33)	4,049 (60.58)	3,895 (59.70)	823 (62.40)		4,286 (64.42)	3,157 (59.11)	1,309 (52.21)		3,227 (63.03)	4,519 (59.26)	1,021 (57.33)	
Quit, *n* (%)	1,292 (8.89)	592 (8.86)	580 (8.89)	120 (9.10)		462 (6.94)	564 (10.56)	261 (10.41)		432 (8.44)	675 (8.85)	185 (10.39)	
Current, *n* (%)	4,468 (30.75)	2,043 (30.57)	2,049 (31.41)	376 (28.51)		1,905 (28.63)	1,620 (30.33)	937 (37.38)		1,461 (28.54)	2,432 (31.89)	575 (32.29)	
Drinking status (missing = 3,641)					0.0873				< 0.0001				0.0585
Never, *n* (%)	8,541 (58.77)	3,904 (77.82)	3,835 (79.12)	802 (78.09)		4,239 (81.68)	2,996 (75.89)	1,289 (74.42)		3,111 (79.12)	4,427 (78.41)	1,003 (76.39)	
Quit, *n* (%)	1,167 (8.03)	556 (11.08)	484 (9.99)	127 (12.37)		472 (9.09)	463 (11.73)	230 (13.28)		410 (10.43)	587 (10.40)	170 (12.95)	
Current, *n* (%)	1,183 (8.14)	557 (11.10)	528 (10.89)	98 (9.54)		479 (9.23)	489 (12.39)	213 (12.30)		411 (10.45)	632 (11.19)	140 (10.66)	
Physical activity (missing = 2,360)					0.4266				0.0069				0.1229
Walking, *n* (%)	10,508 (72.31)	4,819 (72.09)	4,707 (72.10)	982 (74.45)		4,748 (71.33)	3,868 (72.41)	1,870 (74.56)		3,662 (71.51)	5,521 (72.37)	1,325 (74.35)	
Moderate, *n* (%)	1,873 (12.89)	875 (13.09)	837 (12.82)	161 (12.21)		856 (12.86)	704 (13.18)	313 (12.48)		692 (13.51)	960 (12.58)	221 (12.40)	
Vigorous, *n* (%)	2,151 (14.8)	991 (14.82)	984 (15.07)	176 (13.34)		1,052 (15.81)	770 (14.41)	325 (12.96)		767 (14.98)	1,148 (15.05)	236 (13.24)	
Mean nighttime sleep duration, h	6.69 (1.57)	5.30 (0.78)	7.52 (0.50)	9.68 (0.84)	< 0.0001	6.59 (1.59)	6.67 (1.47)	7.04 (1.66)	< 0.0001	5.13 (0.79)	7.19 (0.88)	9.05 (1.17)	< 0.0001
Mean total sleep duration, h	7.35 (1.81)	5.88 (1.08)	8.22 (0.92)	10.46 (1.27)	< 0.0001	6.59 (1.59)	7.51 (1.51)	9.01 (1.73)	< 0.0001	5.44 (0.78)	7.89 (0.74)	10.50 (0.99)	
Mean daytime napping, min	39.21 (44.75)	34.94 (42.33)	42.05 (45.34)	46.79 (51.24)	< 0.0001	0.00 (0.00)	50.94 (14.51)	118.26 (22.65)	< 0.0001	18.67 (29.76)	41.86 (42.08)	86.80 (51.98)	< 0.0001
**Clinical/biochemical measures**													
BMI ≥ 25, *n* (%)	3,810 (26.22)	1,679 (25.12)	1,826 (27.97)	305 (23.12)	< 0.0001	1,550 (23.29)	1,511 (28.29)	740 (29.51)	< 0.0001	1,218 (23.78)	2,107 (27.62)	485 (27.22)	< 0.0001
Elevated waist circumference, *n* (%)	6,213 (42.75)	2,816 (50.12)	2,829 (51.61)	568 (51.87)	0.2338	2,785 (50.13)	2,300 (51.51)	1,117 (51.83)	0.2535	2,146 (50.41)	3,269 (50.86)	798 (52.85)	0.2601
Elevated blood pressure, *n* (%)	6,422 (44.19)	2,945 (44.07)	2,898 (44.41)	579 (43.93)	0.9049	2,863 (43.03)	2,346 (43.93)	1,203 (47.99)	0.0001	2,213 (43.23)	3,383 (44.36)	826 (46.38)	0.0656
Elevated triglycerides, *n* (%)	3,140 (21.61)	1,415 (21.29)	1,441 (22.21)	284 (21.60)	0.4442	1,330 (20.09)	1,226 (23.06)	578 (23.24)	0.0001	1,063 (20.88)	1,668 (21.98)	409 (23.09)	0.1129
Reduced HDL-C, *n* (%)	4,402 (30.29)	2,002 (30.13)	1,989 (30.65)	411 (31.25)	0.6553	1,942 (29.33)	1,658 (31.19)	795 (31.97)	0.0185	1,526 (29.98)	2,313 (30.48)	563 (31.79)	0.3619
Elevated fasting glucose, *n* (%)	5,470 (37.64)	2,574 (54.01)	2,424 (52.15)	472 (50.70)	0.0743	2,436 (52.04)	2,078 (53.57)	947 (53.62)	0.2924	1,965 (53.84)	2,843 (52.37)	662 (52.29)	0.3521
Metabolic syndrome, *n* (%)	4,094 (28.17)	1,845 (27.60)	1,880 (28.80)	369 (27.98)	0.3046	1,748 (26.26)	1,585 (29.67)	755 (30.10)	< 0.0001	1,384 (27.03)	2,192 (28.73)	518 (29.07)	0.0737
**Clinical characteristics**													
Health state (missing = 1)					< 0.0001				0.0617				< 0.0001
Good, *n* (%)	504 (4.54)	169 (3.17)	287 (6.01)	48 (4.86)		232 (4.55)	181 (4.46)	91 (4.74)		126 (3.06)	304 (5.38)	74 (5.58)	
Fair, *n* (%)	6,964 (62.74)	3,220 (60.31)	3,132 (65.61)	612 (62.01)		3,140 (61.54)	2,618 (64.47)	1,195 (62.30)		2,426 (58.87)	3,726 (65.90)	812 (61.28)	
Poor, *n* (%)	3,632 (32.72)	1,950 (36.52)	1,355 (28.38)	327 (33.13)		1,730 (33.91)	1,262 (31.08)	632 (32.95)		1,569 (38.07)	1,624 (28.72)	439 (33.13)	
History of stroke, *n* (%) (missing = 112)	356 (2.44)	180 (2.70)	142 (2.18)	39 (2.96)	0.0835	150 (2.26)	136 (2.55)	74 (2.96)	0.1449	148 (2.89)	155 (2.04)	58 (3.26)	0.0008
History of heart problems, *n* (%) (missing = 74)	1,723 (11.86)	912 (13.72)	690 (10.62)	121 (9.23)	< 0.0001	709 (10.69)	716 (13.48)	294 (11.81)	< 0.0001	718 (14.09)	820 (10.80)	185 (10.45)	< 0.0001
History of cancer, *n* (%) (missing = 62)	144 (0.99)	83 (1.25)	49 (0.75)	12 (0.91)	0.016	64 (0.96)	58 (1.09)	22 (0.88)	0.6373	63 (1.24)	66 (0.87)	15 (0.85)	0.0979
History of disabilities, *n* (%)	2,358 (16.23)	1,125 (16.83)	952 (14.58)	281 (21.30)	< 0.0001	1,015 (15.25)	863 (16.15)	474 (18.90)	0.0001	849 (16.58)	1,158 (15.18)	351 (19.70)	< 0.0001
History of psychiatric problem, *n* (%) (missing = 57)	149 (1.03)	82 (1.23)	55 (0.85)	12 (0.91)	0.0827	60 (0.90)	55 (1.03)	34 (1.36)	0.1532	67 (1.31)	61 (0.80)	21 (1.19)	0.0157
History of memory related disease (missing = 43)	185 (1.27)	95 (1.42)	66 (1.01)	24 (1.83)	0.0194	75 (1.13)	67 (1.26)	43 (1.72)	0.0811	67 (1.31)	86 (1.13)	32 (1.80)	0.0726

Data are % for categorical variables and mean (SD) for continuous variables.

*P*-values were derived from analysis of variance or Mann-Whitney U tests for continuous variables according to data distribution and χ2 tests for category variable.

BMI, body mass index; HDL-C, high-density lipoprotein cholesterol.

### Sleep duration and stroke events

**FIGURE 2:**
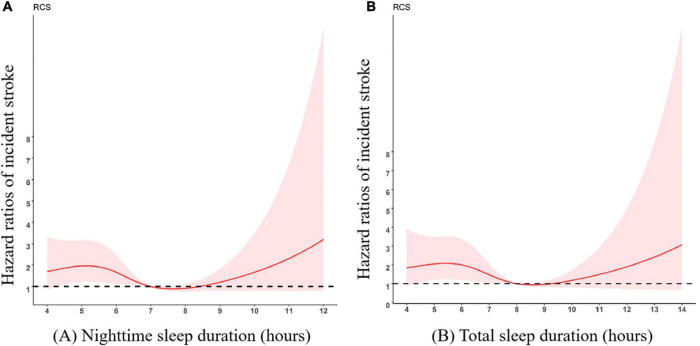
Multivariate-adjusted spline curves for associations of sleep duration with incident stroke. The curves demonstrate that participants with short nighttime sleep duration **(A)** and total sleep duration **(B)** had a higher risk of stroke. **(A)** Nighttime sleep duration; **(B)** Total sleep duration. Adjusted for age, gender, educational level, marital status, area of residence, and behaviors including smoking, drinking, and physical activity. The reference group was 7 h/night for nighttime sleep duration and 8 h/day for total sleep duration.

The cross-sectional relationship between sleep parameters and the stroke risk are presented in Table 2. There was a significant association between short or long total sleep duration and stroke in the crude model, for total sleep duration < 7 h [Odds ratio [OR] (95%CI), 1.43 (1.14–1.80)] and for total sleep duration > 9 h [OR (95%CI), 1.62 (1.20–2.21)]. During a median 4 years of follow-up, 136 (1.98%) (77 cases with short sleep duration, 10 cases with long sleep duration) of the participants experienced incident stroke events. Compared with sleeping for 6∼8 h/night, the multivariate-adjusted HRs (95%CIs) of incident stroke were 1.65 (1.04–2.61) for < 6 h per night. Participants who slept < 7 h in total had an increased risk of developing stroke compared to participants who slept 7∼9 h per day (HRs 1.62 [95%CIs 1.03–2.53]), with adjustment for each of covariates, the significant association was still present. However, there were no significant associations between long sleep duration and incident stroke; nor were there any significant association between napping and incident stroke. The HRs (95%CIs) for each of the covariates in the multivariate-adjusted models are presented in Table 3. We further observed a non-linear association between sleep duration per night/day and incident stroke risk, and an approximately smoothing “U-shape” of such association was showed in Figure 2 and Supplementary Figure 1, in which the lowest risk was observed in participants with sleep duration around 7 h.

**TABLE 2:** The association of sleep duration and daytime napping with stroke.

	Total stroke, adjusted OR (95%CI)			
Variable	Univariate model	Age and gender adjusted	Multivariable adjusted^†^	Multivariable adjusted^‡^
**Nighttime sleep duration, hours/night**				
≤ 6 h	1.24 (0.99, 1.55)	1.17 (0.93, 1.46)	1.16 (0.91, 1.49)	0.95 (0.69, 1.31)
6 to ≤ 8 h	1.000	1.000	1.000	1.000
>8 h	1.37 (0.96, 1.96)	1.23 (0.85, 1.76)	1.07 (0.71, 1.62)	0.65 (0.35, 1.21)
**Daytime napping, minutes**				
0	0.88 (0.70, 1.12)	0.94 (0.74, 1.19)	0.82 (0.63, 1.07)	0.73 (0.51, 1.04)
1 to ≤60 min	1.000	1.000	1.000	1.000
>60 min	1.17 (0.87, 1.55)	1.09 (0.82, 1.46)	1.03 (0.74, 1.42)	1.10 (0.73, 1.65)
**Total sleep duration, hours/day**				
<7 h	**1.43 (1.14, 1.80)** **	1.39 (1.11, 1.75)**	**1.34 (1.04, 1.72)** *	1.08 (0.77, 1.51)
7 to ≤9 h	1.000	1.000	1.000	1.000
>9 h	**1.62 (1.20, 2.21)** **	**1.46 (1.07, 1.99)** *	1.27 (0.89, 1.81)	1.09 (0.68, 1.75)

OR, odds ratio; CI, confidence interval.

*P < 0.05; **P < 0.01.

^†^Adjusted for age, gender, educational level, marital status, area of residence, and behaviors including smoking, drinking, and physical activity.

^‡^Adjusted for age, gender, educational level, marital status, area of residence, behaviors including smoking, drinking, and physical activity, along with additional adjustment for self-reported diagnosis of stroke, dyslipidemia, hypertension, diabetes or high blood sugar, BMI, waist circumference, triglycerides, HDL-C, systolic and diastolic blood pressure, fasting plasma glucose and HbA1c levels.

Bold values indicate the positive results of the study.

**TABLE 3:** Hazard ratios (95%CI) of incident stroke by sleep duration and daytime napping.

Variable	N case/total	Incidence rates,% (95%CI)	Incident stroke, adjusted HR (95%CI)			
			Univariate model	Age and gender adjusted	Multivariable adjusted^†^	Multivariable adjusted^‡^
**Nighttime sleep duration, hours/night**						
≤6 h	77/3,206	2.40 (1.90,2.99)	**1.51 (1.05, 2.16)** *	1.41 (0.98, 2.02)	**1.65 (1.04, 2.61)** *	**1.65 (1.04, 2.61)** *
6 to ≤8 h	49/3,065	1.60 (1.19, 2.11)	1.000	1.000	1.000	1.000
>8 h	10/606	1.65 (0.79,3.01)	1.04 (0.52, 2.04)	1.02 (0.52, 2.01)	1.39 (0.65, 2.94)	1.38 (0.65, 2.93)
**Daytime napping, minutes**						
0	66/3,148	2.10 (1.63, 2.66)	1.11 (0.77, 1.61)	1.18 (0.81, 1.73)	1.36 (0.85, 2.16)	1.36 (0.86, 2.18)
1 to ≤60 min	48/2,543	1.89 (1.39, 2.49)	1.000	1.000	1.000	1.000
>60 min	22/1,186	1.85 (1.17, 2.80)	0.98 (0.59, 1.63)	0.90 (0.54, 1.51)	0.94 (0.49, 1.82)	0.94 (0.49, 1.82)
**Total sleep duration, hours/day**						
<7 h	62/2,443	2.54 (1.95, 3.24)	**1.53 (1.07, 2.18)** *	**1.52 (1.06, 2.18)** *	**1.61 (1.03, 2.52)** *	**1.62 (1.03, 2.53)** *
7 to ≤9 h	60/3,598	1.67 (1.27, 2.14)	1.000	1.000	1.000	1.000
>9 h	14/836	1.67 (0.92, 2.79)	1.01 (0.56, 1.80)	0.98 (0.54, 1.75)	1.11 (0.56, 2.18)	1.11 (0.56, 2.17)

HR, hazard ratio; CI, confidence interval. *P < 0.05.

^†^Adjusted for age, gender, educational level, marital status, area of residence, and behaviors including smoking, drinking, and physical activity.

^‡^Adjusted for age, gender, educational level, marital status, area of residence, behaviors including smoking, drinking, and physical activity, along with additional adjustment for self-reported diagnosis of stroke, dyslipidemia, hypertension, diabetes or high blood sugar, BMI, waist circumference, triglycerides, HDL-C, systolic and diastolic blood pressure, fasting plasma glucose and HbA1c levels.

Bold values indicate the positive results of the study.

### Combined effects of nightly sleep duration and daytime napping on the risks of incident stroke

**FIGURE 3:**
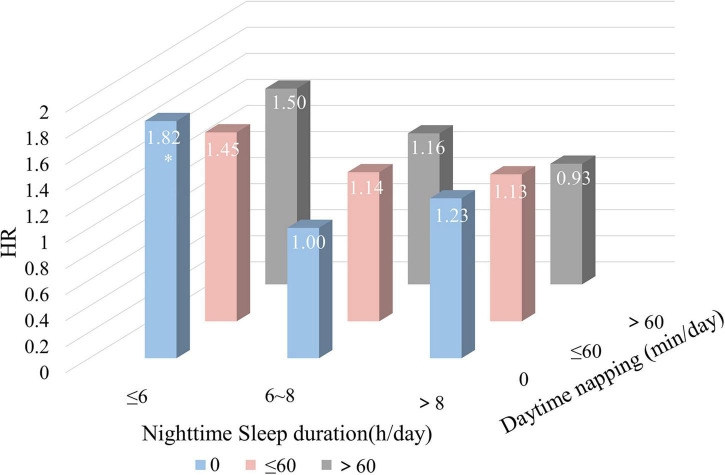
Combined effects of nighttime sleep duration and daytime napping on the risk of stroke. All hazard ratios were calculated with moderate sleep duration (6∼8 h/night) and no napping as the reference groups. HR, hazard ratio. *P < 0.05.

We further explored the joint effects of nighttime sleep duration and daytime napping on the risks of incident stroke. Compared with those reporting moderate nighttime sleep duration (6∼8 h/night) and no napping, participants who reported both short sleep duration (≤ 6 h/night) and no napping showed a significantly higher risk of incident stroke (HR 1.82, 95%CI 1.06–3.12), whereas participants with nighttime sleep ≤ 6 h combined with daytime napping > 60 min were linked with a non-significant risk of stroke. Moreover, our results revealed that long sleep duration (>8 h/night) with different napping patterns had no significant joint effect on stroke risk (Figure 3 and Supplementary Table 1).

### Subgroup analysis

**FIGURE 4:**
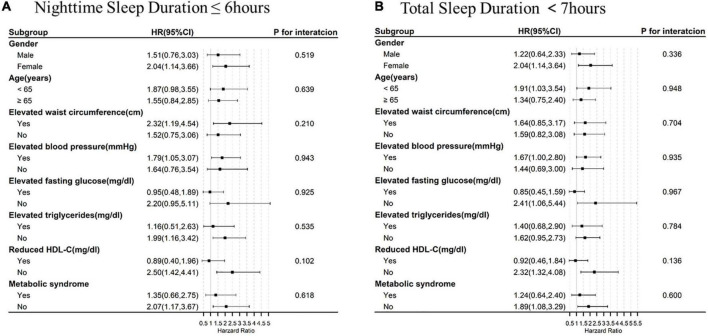
Sleep duration and incident stroke risk, stratified by baseline characteristics and metabolic syndrome. All hazard ratios were calculated with **(A)** night sleep duration > 6 h/night and **(B)** total sleep duration ≥ 7 h/day as the reference groups, with models adjusted for gender, age, educational level, marital status, area of residence, behaviors including smoking, drinking, and physical activity, along with additional adjustment for self-reported diagnosis of stroke, dyslipidemia, hypertension, diabetes or high blood sugar, BMI, waist circumference, triglycerides, HDL-C, systolic and diastolic blood pressure, fasting plasma glucose, and HbA1c levels. Each group adjusted for the other covariates except itself. HR, hazard ratio; CI, confidence interval.

In the subgroup analysis, the association of short sleep duration (<7 h) with incident stroke was more conspicuous among individuals who were female or aged 45–65 years. But the effect tended to attenuate in individuals with nighttime sleep duration ≤ 6 h after adjustment for the potential confounders. When stratified by Mets and its components, the association of incident stroke with short nighttime sleep duration seemed to be more pronounced among individuals with elevated waist circumference and elevated blood pressure, but no interaction was observed. However, for participants with elevated fasting glucose, elevated triglycerides, reduced HDL-C and Mets, the risk of stroke was not significantly different between the different sleep duration groups. The association of short total sleep duration (<7 h) with prevalent stroke had similar trend, but no significant interaction was found (Figure 4 and Supplementary Figure 2). In the sensitivity analysis, the results seemed to show the same tendency (Supplementary Tables 2, 3).

## Discussion

In this prospective cohort study, we found that both short nighttime sleep duration and total sleep duration were significantly associated with higher risk of incident stroke, and a U-shaped of such association was observed. In addition, we observed that short nighttime sleep duration combined with no napping had the highest risk of stroke; proper napping could lower the risk of stroke among those with short nighttime sleep duration. Furthermore, we found that the association of short sleep duration and stroke was more evident in female, those aged 45–65 years and participants with some components of Mets, especially elevated blood pressure.

Stroke is a common and frequently-occurring disease among the elderly, and the absolute numbers and crude rate of stroke burden increased over a 30-year period from 1990 to 2019 (2). In previous studies, the cumulative incidence of stroke was 2.22% in a Chinese study with 8-year follow-up (17), versus 2.75% in the REGARDS study including 16,733 participants with mean follow-up of 6.1 years (36). Similarly, in CHARLS cohort including 6,877 Chinese participants aged 45–90 years with an average follow-up of 4 years, 136 (1.98%) stroke events occurred. In this study, the incidence risk of stroke is relatively low, possibly because of the relative younger sample (71.51% aged younger than 65) in our cohort, as well as the relative shorter follow-up.

Up to date, the impact of sleep duration on stroke risk was inconclusive. Several studies reported a J-shape relationship between short (less than 6 h) or long sleep (more than 9 h) duration with stroke (5–37), while other studies reported a U-shape relationship (4–13). In our study, the cross-sectional study showed a U-shaped association between total sleep duration (night sleep combined with napping) and risk of stroke, indicating the less nighttime sleep can be compensated by napping to get sufficient sleep. Furthermore, in the cohort study, we found a J-shaped association between short sleep duration and high stroke risk. Present results are partially consistent with the preponderance of previous studies. Different age samples and sleep duration compositions may be account for the inconsistency. Besides, time sequence and duration of follow-up may partially explain the differences. The mechanisms underlying the associations between short sleep duration and incident stroke are likely multifactorial. Inflammation, as a previous study shown that sleep deprivation resulted in endothelial dysfunction and elevated markers of oxidative stress and inflammation in blood (38), is a potential biological pathway. Moreover, previous studies showed that habitually short sleep duration was significantly associated with reduced leptin and elevated ghrelin levels (39), increased caloric intake and unhealthy food choices (40, 41), any of which could promote the development of obesity. Short sleep duration may increase plasma cortisol levels via activation of sympathetic activity and the hypothalamic-pituitary-adrenal axis, which can further result in hypertension (42–45). Short sleep duration also results in impaired glucose regulation, insulin resistance (43–46), and hypercholesteremia (47). These common risk factors of cardiovascular events are apt to promote the development of stroke.

Furthermore, a significant joint effect of sleep duration and napping on incident stroke were observed in this study: short sleep duration combined with no napping showed a significantly highest risk of incident stroke, whereas once short sleep duration combined with a daytime napping < 1 h, the risk of stroke relatively lowered. Several studies have provided well-documented evidence of the benefits of naps during total sleep deprivation (48). And there is general agreement that short night sleep duration and no napping could have adverse health consequences with respect to stroke incidence and optimal daytime napping could buffer the adverse effects especially of short night sleep duration.

In addition, we found that the association between short sleep duration and the risk of incident stroke appeared to be more pronounced in participants aged 45–65 years, which is consistent with previous literature (6–49). Furthermore, our study found that women with a sleep duration < 7 h/day had a higher risk for stroke than men. This is in line with a previous study showing that short sleep duration imparted a greater risk of stroke among young women (18–44 years) compared to young men with brief sleep (49). However, the mechanisms are still under exploration.

Among the participants with Mets or its components, the relationship between short sleep duration per day/night and stroke was inconsistent. The risk of stroke seemed to be more evident among those suffering from elevated blood pressure. In line with present results, several previous studies have also reported an association between sleep duration and stroke risk in hypertensive patients (31, 50). The possibility may be that hypertension and short sleep duration have a synergistic effect on stroke risk through common pathways inducing arterial stiffening and atherosclerosis. In addition, we suppose sleep duration could lead to increased blood pressure and prevalence of hypertension, which, in turn, might lead to prevalence of stroke. This finding suggests that optimal sleep duration is particularly important for hypertensive patients. However, the association of short sleep duration and incident stroke is not statistically significant in participants with hyperlipidemia, elevated blood glucose or metabolic syndrome, while in persons relatively free of hyperlipidemia, diabetes and Mets, short sleep duration itself may be a precursor to other traditional stroke risk factors, which exacts its own negative influence on stroke. The results presented in our study are in agreement with previous studies (51–54). Cross-sectional studies indicated short sleep duration was not associated with the increased risk of stroke among individuals with Mets (52) and Asian diabetic patients (53). Moreover, a longitudinal study confirmed that only in individuals of normal weight, self-reported short sleep duration had an increased risk of stroke symptoms (54). However, there is little known about the underlying mechanism in the association between sleep duration and stroke and the mediating role of Mets, which need to be further validated in future studies.

### Strengths and limitations

Our analysis had several strengths. Most notably, this study explored the association of sleep duration and incident stroke among participants with components of metabolic syndrome, as issue rarely considered in prior studies. Then, the study benefited from its prospective design and involvement of a large, Chinese population-based sample of middle-aged to older adults. Third, samples were obtained by random sampling method and multiple stroke related factors assessed through validated screening questionnaire. Thus, the population was representative, and the measurement method was objective. Finally, we both conducted cross-sectional and longitudinal study to explore the association between sleep duration and risk of stroke, which likely increased the accuracy of the results.

However, some limitations also should be noted. First, information on sleep duration and midday napping was obtained from a questionnaire rather than by recording biological sleep. Although prone to biases in perception, self-report is a convenient and frequently used method to assess sleep duration and napping in numerous large population-based studies (5, 17, 36). Moreover, questionnaire could reflect their own understanding and assessment of sleep conditions. Second, we did not collect information on sleep disorders and dimensions such as snoring, sleep apnea, sleep quality, bedtime and wake-up time, any of which might be related to the risk of stroke according to previous studies (5, 55, 56). Our further research will focus on collecting data about sleep dimensions and consider the contribution of these confounding factors. Finally, in our study, participants with long sleep duration accounted for only 9.1%, the number of which may be relatively small to explore the reliable relationship between long sleep duration and stroke risk. Therefore, the data on the right half of our U-shape may be insufficient for the discussion of long sleep and further studies should be confirmed.

## Conclusion

Our prospective study revealed that short sleep duration is a significant, independent risk predictor of stroke incidence, especially among females adults aged 45–65 years. Furthermore, the effect was different among Mets and its components, more evident among those with hypertension, but lower in those with Mets, elevated triglycerides and reduced HDL-C. Moreover, optimal daytime sleeping could compensate the adverse effects of short nighttime sleep duration on stroke. Our results highlight the importance of adequate sleep duration for stroke prevention, and the need for better understanding of the role of Mets on sleep-stroke association.

## Data availability statement

The original contributions presented in this study are included in the article/Supplementary material, further inquiries can be directed to the corresponding authors.

## Author contributions

YF: conceptualization, data extraction, methodology, software, and writing original draft—participated in all aspects of this research. TQ: data curation, formal analysis, and revised the manuscript. YQH, LR, and WS: data extraction and revised the manuscript. JH, DY, and RL: retrieval literature—original draft preparation. DP and YZH: conceptualization, reviewing, and supervision. MW: conceptualization, methodology, revising—reviewing and editing, and supervision. All authors contributed to the article and approved the submitted version.

## Abbreviations

CHARLS: China health and retirement longitudinal study
Mets: metabolic syndrome
BMI: body mass index
HDL-C: high-density lipoprotein cholesterol
DALYs: death and disability-adjusted life-years
HR: hazard ratio
OR: odds ratio
95%CI: 95% confidence interval.

## References

[1] Zhou M Wang H Zeng X Yin P Zhu J Chen W Mortality, morbidity, and risk factors in China and its provinces, 1990–2017: a systematic analysis for the Global Burden of Disease Study 2017. *Lancet.* (2019) 394:1145–58. 10.1016/S0140-6736(19)30427-1 31248666 PMC6891889

[2] Ma Q Li R Wang L Yin P Wang Y Yan C Temporal trend and attributable risk factors of stroke burden in China, 1990–2019: an analysis for the Global Burden of Disease Study 2019. *Lancet Public Health.* (2021) 6:e897–906. 10.1016/S2468-2667(21)00228-0 34838196 PMC9047702

[3] Wang J Wen X Li W Li X Wang Y Lu W. Risk factors for stroke in the Chinese population: a systematic review and meta-analysis. *J Stroke Cerebrovasc Dis.* (2017) 26:509–17. 10.1016/j.jstrokecerebrovasdis.2016.12.002 28041900

[4] Zhu G Cassidy S Hiden H Woodman S Trenell M Gunn DA Exploration of sleep as a specific risk factor for poor metabolic and mental health: a UK biobank study of 84,404 participants. *Nat Sci Sleep.* (2021) 13:1903. 10.2147/NSS.S323160 34712066 PMC8548259

[5] Zhou L Yu K Yang L Wang H Xiao Y Qiu G Sleep duration, midday napping, and sleep quality and incident stroke: the Dongfeng-Tongji cohort. *Neurology.* (2020) 94:e345–56. 10.1212/WNL.0000000000008739 31827003

[6] Hirshkowitz M Whiton K Albert SM Alessi C Bruni O DonCarlos L National Sleep Foundation’s updated sleep duration recommendations: final report. *Sleep Health.* (2015) 1:233–43. 10.1016/j.sleh.2015.10.004 29073398

[7] Cappuccio FP Taggart FM Kandala NB Currie A Peile E Stranges S Meta analysis of short sleep duration and obesity in children and adults. *Sleep.* (2008) 31:619–26. 10.1093/sleep/31.5.619 18517032 PMC2398753

[8] Gottlieb DJ Punjabi NM Newman AB Resnick HE Redline S Baldwin CM Association of sleep time with diabetes mellitus and impaired glucose tolerance. *Archintern Med.* (2005) 165:863–7. 10.1001/archinte.165.8.863 15851636

[9] Knutson KL Van Cauter E Rathouz PJ Yan LL Hulley SB Liu K Association between sleep and blood pressure in midlife: the CARDIA sleep study. *Arch Intern Med.* (2009) 169:1055–61. 10.1001/archinternmed.2009.119 19506175 PMC2944774

[10] Adedayo AM Olafiranye O Smith D Hill A Zizi F Brown C Obstructive sleep apnea and dyslipidemia: evidence and underlying mechanism. *Sleep Breath.* (2014) 18:13–8. 10.1007/s11325-012-0760-9 22903801 PMC4805366

[11] Kim M-Y Lee S Myong YH Lee YJ Kim M-R Shin J-S Association between sleep duration and stroke prevalence in Korean adults: a cross-sectional study. *BMJ Open.* (2018) 8:e021491. 10.1136/bmjopen-2018-021491 29903797 PMC6009631

[12] Jike M Itani O Watanabe N Buysse DJ Kaneita Y. Long sleep duration and health outcomes: a systematic review, meta-analysis and meta-regression. *Sleep Med Rev.* (2018) 39:25–36. 10.1016/j.smrv.2017.06.011 28890167

[13] Titova OE Michaëlsson K Larsson SC. Sleep duration and stroke: prospective cohort study and mendelian randomization analysis. *Stroke.* (2020) 51:3279–85. 10.1161/STROKEAHA.120.029902 32895015 PMC7587241

[14] Chen J-C Brunner RL Ren H Wassertheil-Smoller S Larson JC Levine DW Sleep duration and risk of ischemic stroke in postmenopausal women. *Stroke.* (2008) 39:3185–92. 10.1161/STROKEAHA.108.521773 18635832 PMC2587518

[15] Li W Wang D Cao S Yin X Gong Y Gan Y Sleep duration and risk of stroke events and stroke mortality: a systematic review and meta-analysis of prospective cohort studies. *Int J Cardiol.* (2016) 223:870–6. 10.1016/j.ijcard.2016.08.302 27584562

[16] He Q Sun H Wu X Zhang P Dai H Ai C Sleep duration and risk of stroke: a dose–response meta-analysis of prospective cohort studies. *Sleep Med.* (2017) 32:66–74. 10.1016/j.sleep.2016.12.012 28366344

[17] Ji A Lou H Lou P Xu C Zhang P Qiao C Interactive effect of sleep duration and sleep quality on risk of stroke: an 8-year follow-up study in China. *Sci Rep.* (2020) 10:1–9. 10.1038/s41598-020-65611-y 32457400 PMC7250859

[18] Westerlund A Bellocco R Sundström J Adami H-O Åkerstedt T Lagerros YT. Sleep characteristics and cardiovascular events in a large Swedish cohort. *Eur J Epidemiol.* (2013) 28:463–73. 10.1007/s10654-013-9802-2 23553209

[19] Itani O Jike M Watanabe N Kaneita Y. Short sleep duration and health outcomes: a systematic review, meta-analysis, and meta-regression. *Sleep Med.* (2017) 32:246–56. 10.1016/j.sleep.2016.08.006 27743803

[20] Lin D Sun K Li F Qi Y Ren M Huang C Association between habitual daytime napping and metabolic syndrome: a population-based study. *Metabolism.* (2014) 63:1520–7. 10.1016/j.metabol.2014.08.005 25249445

[21] Li J Cacchione PZ Hodgson N Riegel B Keenan BT Scharf MT Afternoon napping and cognition in Chinese older adults: findings from the china health and retirement longitudinal study baseline assessment. *J Am Geriatr Soc.* (2017) 65:373–80. 10.1111/jgs.14368 27995615 PMC6487643

[22] Yan B Jin X Li R Gao Y Zhang J Li J Association of daytime napping with incident stroke in middle-aged and older adults: a large community-based study. *Eur J Neurol.* (2020) 27:1028–34. 10.1111/ene.14197 32129913

[23] Li X Pang X Liu Z Zhang Q Sun C Yang J Joint effect of less than 1 h of daytime napping and seven to 8 h of night sleep on the risk of stroke. *Sleep Med.* (2018) 52:180–7. 10.1016/j.sleep.2018.05.011 30408698

[24] Gupta AK Dahlof B Sever PS Poulter NR. Anglo-scandinavian cardiac outcomes trial-blood pressure lowering arm I. Metabolic syndrome, independent of its components, is a risk factor for stroke and death but not for coronary heart disease among hypertensive patients in the ASCOT-BPLA. *Diabetes Care.* (2010) 33:1647–51. 10.2337/dc09-2208 20413525 PMC2890375

[25] Rodriguez-Colon SM Mo J Duan Y Liu J Caulfield JE Jin X Metabolic syndrome clusters and the risk of incident stroke: the atherosclerosis risk in communities (ARIC) study. *Stroke.* (2009) 40:200–5. 18927451 10.1161/STROKEAHA.108.523035

[26] Lu J Wang L Li M Xu Y Jiang Y Wang W Metabolic syndrome among adults in China: the 2010 China noncommunicable disease surveillance. *J Clin Endocrinol Metab.* (2017) 102:507–15. 10.1210/jc.2016-2477 27898293

[27] Xie J Li Y Zhang Y Vgontzas AN Basta M Chen B Sleep duration and metabolic syndrome: an updated systematic review and meta-analysis. *Sleep Med Rev.* (2021) 59:101451. 10.1016/j.smrv.2021.101451 33618187

[28] Fan L Hao Z Gao L Qi M Feng S Zhou G. Non-linear relationship between sleep duration and metabolic syndrome: a population-based study. *Medicine.* (2020) 99:e18753. 10.1097/MD.0000000000018753 31914097 PMC6959870

[29] Yamada T Shojima N Yamauchi T Kadowaki T. J-curve relation between daytime nap duration and type 2 diabetes or metabolic syndrome: a dose-response meta-analysis. *Sci Rep.* (2016) 6:1–10. 10.1038/srep38075 27909305 PMC5133463

[30] Xi B He D Zhang M Xue J Zhou D. Short sleep duration predicts risk of metabolic syndrome: a systematic review and meta-analysis. *Sleep Med Rev.* (2014) 18:293–7. 10.1016/j.smrv.2013.06.001 23890470

[31] Eguchi K Hoshide S Ishikawa S Shimada K Kario K. Short sleep duration is an independent predictor of stroke events in elderly hypertensive patients. *J Am Soc Hypertens.* (2010) 4:255–62. 10.1016/j.jash.2010.09.001 20940066

[32] Lin L Lu C Chen W Guo VY. Daytime napping and nighttime sleep duration with incident diabetes mellitus: a cohort study in Chinese older adults. *Int J Environ Res Public Health.* (2021) 18:5012. 10.3390/ijerph18095012 34065152 PMC8125963

[33] Li W Taskin T Gautam P Gamber M Sun W. Is there an association among sleep duration, nap, and stroke? Findings from the China health and retirement longitudinal study. *Sleep Breath.* (2021) 25:315–23. 10.1007/s11325-020-02118-w 32562171

[34] Grundy SM Cleeman JI Daniels SR Donato KA Eckel RH Franklin BA Diagnosis and management of the metabolic syndrome: an American Heart Association/National Heart, Lung, and Blood Institute scientific statement. *Circulation.* (2005) 112:2735–52. 10.1161/CIRCULATIONAHA.105.169404 16157765

[35] Alberti K Eckel RH Grundy SM Zimmet PZ Cleeman JI Donato KA Harmonizing the metabolic syndrome: a joint interim statement of the International Diabetes Federation Task Force on Epidemiology and Prevention; National Heart, Lung, and Blood Institute; American Heart Association; World Heart Federation; International Atherosclerosis Society; and International Association for the Study of Obesity. *Circulation.* (2009) 120:1640–5. 10.1161/CIRCULATIONAHA.109.192644 19805654

[36] Petrov ME Howard G Grandner MA Kleindorfer D Molano JR Howard VJ. Sleep duration and risk of incident stroke by age, sex, and race: the REGARDS study. *Neurology.* (2018) 91:e1702–9. 10.1212/WNL.0000000000006424 30282769 PMC6207412

[37] Von Ruesten A Weikert C Fietze I Boeing H. Association of sleep duration with chronic diseases in the European prospective investigation into cancer and nutrition (EPIC)-potsdam study. *PLoS One.* (2012) 7:e30972. 10.1371/journal.pone.0030972 22295122 PMC3266295

[38] Kröller-Schön S Daiber A Steven S Oelze M Frenis K Kalinovic S Crucial role for Nox2 and sleep deprivation in aircraft noise-induced vascular and cerebral oxidative stress, inflammation, and gene regulation. *Eur Heart J.* (2018) 39:3528–39. 10.1093/eurheartj/ehy333 29905797 PMC6174027

[39] Taheri S Lin L Austin D Young T Mignot E. Short sleep duration is associated with reduced leptin, elevated ghrelin, and increased body mass index. *PLoS Med.* (2004) 1:e62. 10.1371/journal.pmed.0010062 15602591 PMC535701

[40] Brondel L Romer MA Nougues PM Touyarou P Davenne D. Acute partial sleep deprivation increases food intake in healthy men. *Am J Clin Nutr.* (2010) 91:1550–9. 10.3945/ajcn.2009.28523 20357041

[41] Greer SM Goldstein AN Walker MP. The impact of sleep deprivation on food desire in the human brain. *Nat Commun.* (2013) 4:1–7. 10.1038/ncomms3259 23922121 PMC3763921

[42] Vgontzas AN Liao D Bixler EO Chrousos GP Vela-Bueno A. Insomnia with objective short sleep duration is associated with a high risk for hypertension. *Sleep.* (2009) 32:491–7. 10.1093/sleep/32.4.491 19413143 PMC2663863

[43] Spiegel K Leproult R Van Cauter E. Impact of sleep debt on metabolic and endocrine function. *Lancet.* (1999) 354:1435–9. 10.1016/S0140-6736(99)01376-8 10543671

[44] Palagini L Maria Bruno R Gemignani A Baglioni C Ghiadoni L Riemann D. Sleep loss and hypertension: a systematic review. *Curr Pharmaceut Design.* (2013) 19:2409–19. 10.2174/1381612811319130009 23173590

[45] Ceïde ME Pandey A Ravenell J Donat M Ogedegbe G Jean-Louis G. Associations of short sleep and shift work status with hypertension among black and white Americans. *Int J Hypertens.* (2015) 2015:697275. 10.1155/2015/697275 26495140 PMC4606100

[46] Kernan W Inzucchi S Viscoli C Brass L Bravata D Horwitz R. Insulin resistance and risk for stroke. *Neurology.* (2002) 59:809–15. 10.1212/WNL.59.6.809 12349850

[47] Hill A Williams N Salifu I Castor C Gibilaro J. The role of race/ethnicity and gender in the association between inadequate sleep and hypercholesterolemia. *J Sleep Disord Ther.* (2015) 4:1000194.

[48] Milner CE Cote KA. Benefits of napping in healthy adults: impact of nap length, time of day, age, and experience with napping. *J Sleep Res.* (2009) 18:272–81. 10.1111/j.1365-2869.2008.00718.x 19645971

[49] Fang J Wheaton AG Ayala C. Sleep duration and history of stroke among adults from the USA. *J Sleep Res.* (2014) 23:531–7. 10.1111/jsr.12160 24815229 PMC4365417

[50] Pan A De Silva DA Yuan J-M Koh W-P. Sleep duration and risk of stroke mortality among Chinese adults: Singapore Chinese health study. *Stroke.* (2014) 45:1620–5. 24743442 10.1161/STROKEAHA.114.005181 PMC4102627

[51] Sabanayagam C Shankar A. Sleep duration and cardiovascular disease: results from the National Health Interview Survey. *Sleep.* (2010) 33:1037–42. 20815184 10.1093/sleep/33.8.1037 PMC2910533

[52] Ye Y Zhang L Wang A Wang Y Wang S Ning G Association of sleep duration with stroke, myocardial infarction, and tumors in a Chinese population with metabolic syndrome: a retrospective study. *Lipids Health Dis.* (2020) 19:155. 10.1186/s12944-020-01328-1 32593309 PMC7321539

[53] Akinseye OA Ojike NI Akinseye LI Dhandapany PS Pandi-Perumal SR. Association of sleep duration with stroke in diabetic patients: analysis of the National Health Interview Survey. *J Stroke Cerebrovasc Dis.* (2016) 25:650–5. 10.1016/j.jstrokecerebrovasdis.2015.11.023 26738814

[54] Ruiter Petrov ME Letter AJ Howard VJ Kleindorfer D. Self-reported sleep duration in relation to incident stroke symptoms: nuances by body mass and race from the REGARDS study. *J Stroke Cerebrovasc Dis.* (2014) 23:e123–32. 10.1016/j.jstrokecerebrovasdis.2013.09.009 24119626 PMC3946730

[55] Palomäki H Partinen M Erkinjuntti T Kaste M. Snoring, sleep apnea syndrome, and stroke. *Neurology.* (1992) 42(7 Suppl. 6):75–81; discussion 2. 1630643

[56] Nikbakhtian S Reed AB Obika BD Morelli D Cunningham AC Aral M Accelerometer-derived sleep onset timing and cardiovascular disease incidence: a UK Biobank cohort study. *Eur Heart J Digital Health.* (2021) 2:658–66. 10.1093/ehjdh/ztab088 PMC9708010 36713092

