# Supplementary material for: Sleep duration, daytime napping, and risk of incident stroke: Nuances by metabolic syndrome from the China health and retirement longitudinal study
Source: Front Cardiovasc Med. 2022 Sep 2;9:976537. doi: 10.3389/fcvm.2022.976537 (PMC9478414; doi:10.3389/fcvm.2022.976537)
Supplement: Supplementary file 1 [file Data_Sheet_1.doc]

**Supplementary Material**

**Supplementary methods**

The presence of metabolic syndrome was defined by the coexistence of three or more of the following criteria: elevated waist circumference, elevated triglycerides, reduced high-density lipoprotein cholesterol (HDL-C), elevated blood pressure, and elevated fasting glucose (1, 2). In this study, the range of waist circumference was 20-140 cm, whereas plasma triglycerides were in the range of 4-1000 mg/dl, HDL-C of 3-120 mg/dl, systolic blood pressure of 60-300 mmHg, diastolic pressure of 30-150 mmHg, and fasting glucose of 2-450 mg/dl. The protocols for physical measurements and blood collection have been described elsewhere (3, 4).

**Reference**

1. Grundy SM, Cleeman JI, Daniels SR, Donato KA, Eckel RH, Franklin BA, et al. Diagnosis and management of the metabolic syndrome: an American Heart Association/National Heart, Lung, and Blood Institute scientific statement. Circulation. 2005;112(17):2735-52.

2. Alberti K, Eckel RH, Grundy SM, Zimmet PZ, Cleeman JI, Donato KA, et al. Harmonizing the metabolic syndrome: A joint interim statement of the international diabetes federation task force on epidemiology and prevention; National heart, lung, and blood institute; American heart association; World heart federation; International atherosclerosis society; And international association for the study of obesity. Circulation. 2009;120(16):1640-5.

3. Zhao Y, Hu Y, Smith JP, Strauss J, Yang G. Cohort profile: the China health and retirement longitudinal study (CHARLS). International journal of epidemiology. 2014;43(1):61-8.

4. Chen X, Crimmins E, Hu P, Kim JK, Meng Q, Strauss J, et al. Venous blood-based biomarkers in the China health and retirement longitudinal study: rationale, design, and results from the 2015 wave. American journal of epidemiology. 2019;188(11):1871-7.

**Supplementary Figure 1. Multivariate-adjusted spline curves for associations of sleep duration with incident stroke.**


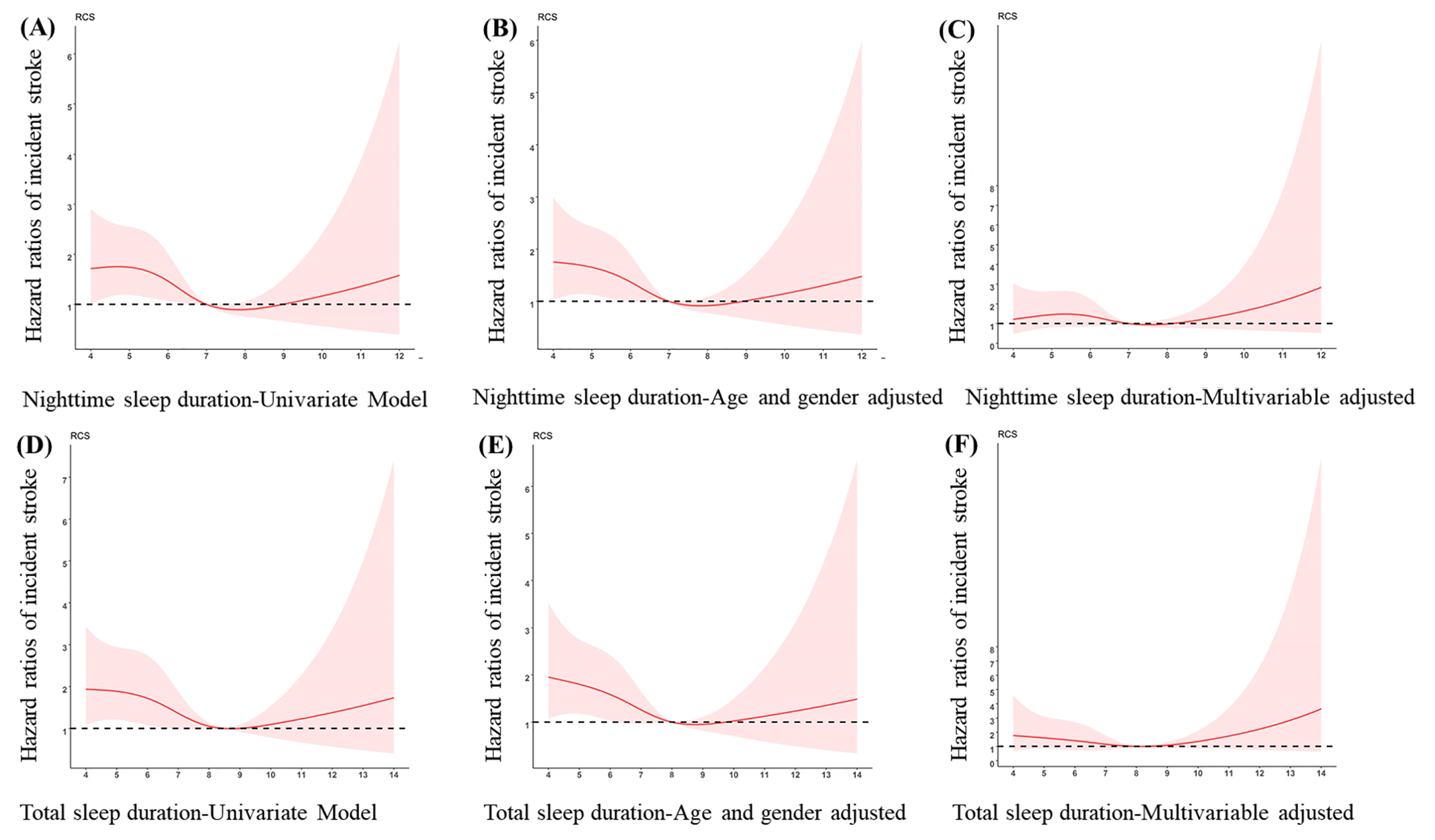


The curves demonstrate that participants with short nighttime sleep duration (A-C) and total sleep duration (D-F) had a higher incidence of stroke. (A-C) Nighttime sleep duration, the reference group was 7 hours/night for sleep duration. (D-F) Total sleep duration, the reference group was 8 hours/day for sleep duration. Multivariable adjusted for age, gender, educational level, marital status, area of residence, behaviors including smoking, drinking, and physical activity, body mass index, self-report diagnosis of stroke, hypertension, hyperlipidemia, diabetes or high blood sugar, waist circumference, systolic and diastolic blood pressure, HDL, triglycerides, fasting plasma glucose and HbA1c.

**Supplementary Figure 2. Multivariate-adjusted association of sleep duration with incident stroke risk, stratified by baseline characteristics and metabolic syndrome.**

**
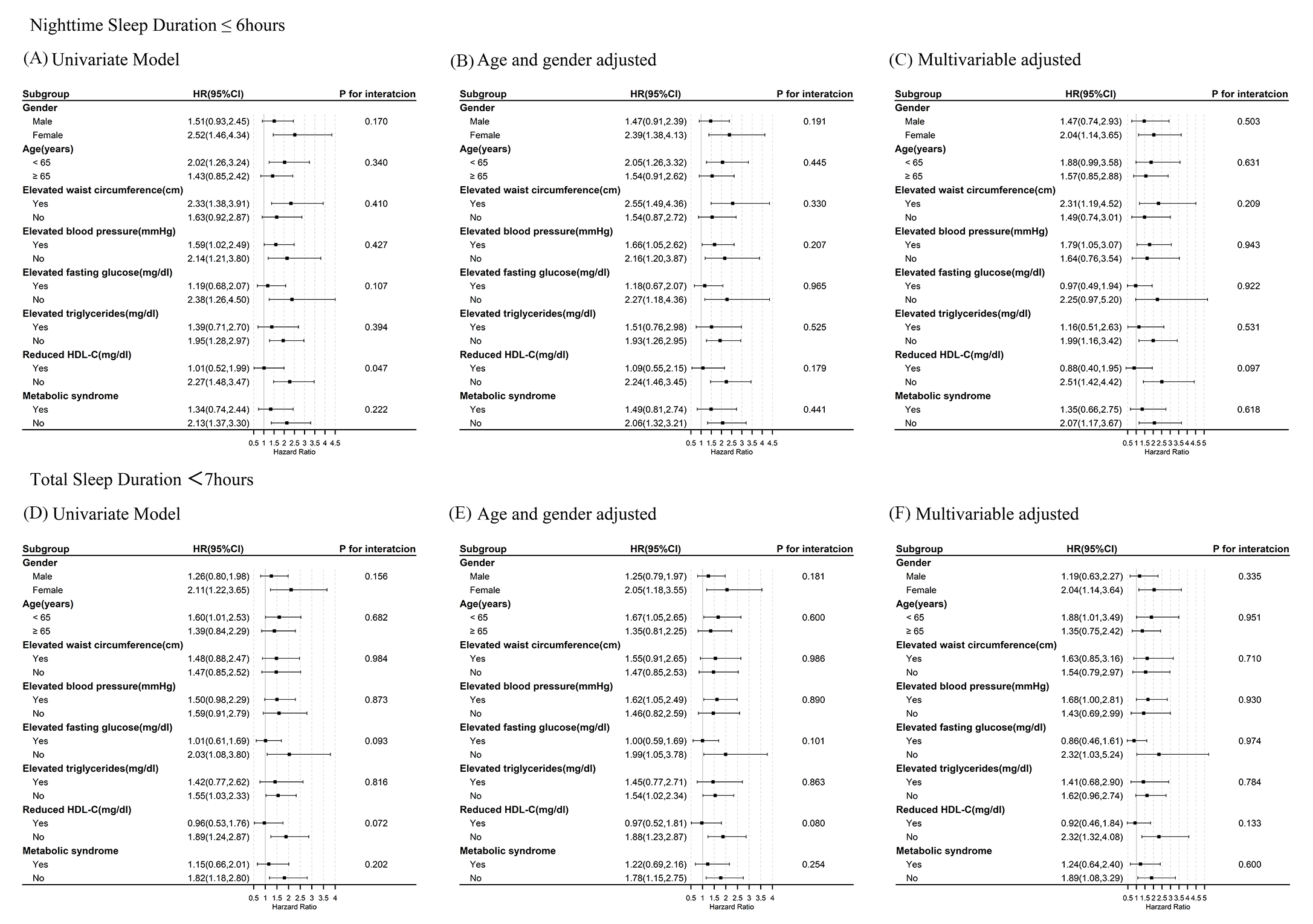
**

All hazard ratios were calculated with night sleep duration >6 hours/night (A-C) and (D-F) total sleep duration ≥7 hours/day as the reference groups. Multivariable model adjusted for age, gender, educational level, marital status, area of residence, and behaviors including smoking, drinking, and physical activity.

Abbreviations: HR = hazard ratio, CI = confidence interval.

**Supplementary Table 1. Joint associations of nighttime sleep duration and daytime napping with incident stroke.**

| **Variable** | | **Incident stroke, Adjusted HR(95% CI)** | | | | |
| --- | --- | --- | --- | --- | --- | --- |
| **Nighttime sleep duration, hours** | **Daytime napping, min** | **cases(N)** | **Univariate Model** | **Age and gender adjusted** | **Multivariable adjusted†** | **Multivariable adjusted‡** |
| N≤6 | D=0 | 42(1579) | **1.82(1.06,3.12)** | 1.71(0.99,2.95) | 1.83(0.95,3.52) | 1.83(0.95,3.52) |
| 6＜N≤8 | D=0 | 19(1291) | 1.00 | 1.00 | 1.00 | 1.00 |
| N＞8 | D=0 | 5(278) | 1.23(0.46,3.29) | 1.21(0.45,3.25) | 1.85(0.66,5.21) | 1.85(0.66,5.20) |
| N≤6 | 0＜D≤60 | 25(1174) | 1.45(0.80,2.63) | 1.26(0.69,2.31) | 1.29(0.62,2.70) | 1.29(0.62,2.69) |
| 6＜N≤8 | 0＜D≤60 | 20(1188) | 1.14(0.61,2.14) | 1.08(0.57,2.02) | 0.94(0.42,2.10) | 0.93(0.41,2.08) |
| N＞8 | 0＜D≤60 | 3(181) | 1.13(0.33,3.81) | 1.04(0.31,3.52) | 0.86(0.19,3.84) | 0.85(0.19,3.79) |
| N≤6 | D＞60 | 10(453) | 1.50(0.70,3.23) | 1.17(0.53,2.58) | 1.31(0.50,3.45) | 1.30(0.49,3.42) |
| 6＜N≤8 | D＞60 | 10(586) | 1.16(0.54,2.492) | 1.02(0.47,2.21) | 0.82(0.29,2.31) | 0.82(0.29,2.30) |
| N＞8 | D＞60 | 2(147) | 0.93(0.22,3.98) | 0.80(0.19,3.45) | 1.05(0.24,4.68) | 1.04(0.23,4.64) |

Abbreviations: HR = hazard ratio; CI = confidence interval. Bold values indicate the positive results of the study.

† adjusted for age, gender, educational level, marital status, area of residence, and behaviors including smoking, drinking, and physical activity;
‡ adjusted for age, gender, educational level, marital status, area of residence, behaviors including smoking, drinking, and physical activity, along with additional adjustment for self-reported diagnosis of stroke, dyslipidemia, hypertension, diabetes or high blood sugar, BMI, waist circumference, triglycerides, HDL-C, systolic and diastolic blood pressure, fasting plasma glucose and HbA1c levels.

| **Supplementary Table 2: Sensitivity analysis: nighttime sleep duration and incident stroke risk, stratified by objectively assessed Mets (N = 4426).** | | | | | | | | | | | | |
| --- | --- | --- | --- | --- | --- | --- | --- | --- | --- | --- | --- | --- |
|  | **Univariate Model** | | | **Age and gender adjusted** | | | **Multivariable adjusted†** | | | **Multivariable adjusted‡** | | |
| Subgroup | HR (95%CI) | P value | P for interaction | HR (95%CI) | P value | P for interaction | HR (95%CI) | P value | P for interaction | HR (95%CI) | P value | P for interaction |
| Elevated waist circumference(cm) |  |  |  |  |  |  |  |  |  |  |  |  |
| Yes | **2.36(1.40,3.96)** | **0.001** | 0.258 | **2.50(1.48,4.23)** | **0.001** | 0.173 | **2.27(1.18,4.34)** | **0.014** | 0.454 | **2.31(1.20,4.42)** | 0.012 | 0.801 |
| No | 1.51(0.86,2.67) | 0.154 |  | 1.43(0.81,2.53) | 0.219 |  | 1.46(0.73,2.93) | 0.288 |  | 1.47(0.73,2.96) | 0.276 |  |
| Elevated blood pressure(mmHg) |  |  |  |  |  |  |  |  |  |  |  |  |
| Yes | **1.61(1.00,2.62)** | **0.052** | 0.327 | **1.72(1.05,2.79)** | **0.030** | 0.334 | **1.78(1.01,3.16)** | **0.048** | 0.903 | 1.70(0.96,3.02) | 0.07 | 0.685 |
| No | **2.42(1.27,4.60)** | **0.007** |  | **2.19(1.15,4.18)** | **0.017** |  | 1.77(0.77,4.07) | 0.177 |  | 1.78(0.78,4.09) | 0.173 |  |
| Elevated fasting glucose(mg/dl) |  |  |  |  |  |  |  |  |  |  |  |  |
| Yes | 1.21(0.62,2.35) | 0.585 | 0.228 | 1.17(0.60,2.30) | 0.643 | 0.188 | 0.99(0.42,2.35) | 0.984 | 0.068 | 1.05(0.44,2.51) | 0.906 | 0.068 |
| No | **2.03(1.21,3.39)** | **0.007** |  | **2.02(1.20,3.39)** | **0.008** |  | **1.87(1.00,3.51)** | **0.050** |  | 1.85(0.99,3.47) | 0.054 |  |
| Elevated triglycerides(mg/dl) |  |  |  |  |  |  |  |  |  |  |  |  |
| Yes | 1.35(0.67,2.71) | 0.400 | 0.495 | 1.35(0.67,2.73) | 0.402 | 0.524 | 0.98(0.41,2.31) | 0.957 | 0.274 | 0.97(0.41,2.31) | 0.951 | 0.294 |
| No | **1.82(1.10,3.01)** | **0.020** |  | **1.78(1.08,2.95)** | **0.025** |  | 1.84(0.98,3.46) | 0.057 |  | 1.82(0.97,3.41) | 0.064 |  |
| Reduced HDL-C(mg/dl) |  |  |  |  |  |  |  |  |  |  |  |  |
| Yes | 0.96(0.48,1.95) | 0.916 | 0.055 | 0.97(0.48,1.97) | 0.933 | 0.071 | 0.76(0.33,1.74) | 0.509 | 0.054 | 0.77(0.33,1.78) | 0.541 | 0.057 |
| No | **2.26(1.36,3.78)** | **0.002** |  | **2.21(1.32,3.70)** | **0.003** |  | **2.50(1.28,4.91)** | **0.008** |  | **2.46(1.25,4.84)** | **0.009** |  |
| Metabolic syndrome |  |  |  |  |  |  |  |  |  |  |  |  |
| Yes | 1.45(0.76,2.77) | 0.261 | 0.501 | 1.52(0.79,2.92) | 0.207 | 0.532 | 1.33(0.60,2.96) | 0.479 | 0.195 | 1.33(0.60,2.96) | 0.479 | 0.351 |
| No | **1.90(1.22,2.95)** | **0.005** |  | **1.83(1.18,2.86)** | **0.008** |  | 1.71(0.99,2.96) | 0.054 |  | 1.71(0.99,2.96) | 0.054 |  |

Abbreviations: HR = hazard ratio; CI = confidence interval. Bold values indicate the positive results of the study.

† adjusted for age, gender, educational level, marital status, area of residence, and behaviors including smoking, drinking, and physical activity;

‡ adjusted for age, gender, educational level, marital status, area of residence, behaviors including smoking, drinking, and physical activity, along with additional adjustment for self-reported diagnosis of stroke, dyslipidemia, hypertension, diabetes or high blood sugar, BMI, waist circumference, triglycerides, HDL-C, systolic and diastolic blood pressure, fasting plasma glucose and HbA1c levels.

| **Supplementary Table 3: Sensitivity analysis: total sleep duration and incident stroke risk, stratified by objectively assessed Mets (N = 4426).** | | | | | | | | | | | | |
| --- | --- | --- | --- | --- | --- | --- | --- | --- | --- | --- | --- | --- |
|  | **Univariate Model** | | | **Age and gender adjusted** | | | **Multivariable adjusted†** | | | **Multivariable adjusted‡** | | |
| Subgroup | HR (95%CI) | P value | P for interaction | HR (95%CI) | P value | P for interaction | HR (95%CI) | P value | P for interaction | HR (95%CI) | P value | P for interaction |
| Elevated waist circumference(cm) |  |  |  |  |  |  |  |  |  |  |  |  |
| Yes | 1.46(0.87,2.44) | 0.151 | 0.956 | 1.60(0.95,2.68) | 0.078 | 0.790 | 1.74(0.92,3.31) | 0.088 | 0.929 | 1.76(0.93,3.35) | 0.084 | 0.909 |
| No | 1.43(0.83,2.45) | 0.223 |  | 1.40(0.82,2.41) | 0.223 |  | 1.52(0.79,2.93) | 0.214 |  | 1.53(0.79,2.96) | 0.2035 |  |
| Elevated blood pressure(mmHg) |  |  |  |  |  |  |  |  |  |  |  |  |
| Yes | 1.35(0.85,2.15) | 0.203 | 0.699 | 1.46(0.92,2.33) | 0.112 | 0.738 | 1.62(0.93,2.83) | 0.090 | 0.960 | 1.55(0.89,2.71) | 0.1243 | 0.997 |
| No | 1.58(0.84,2.99) | 0.160 |  | 1.51(0.80,2.86) | 0.203 |  | 1.53(0.70,3.38) | 0.290 |  | 1.55(0.70,3.42) | 0.2778 |  |
| Elevated fasting glucose(mg/dl) |  |  |  |  |  |  |  |  |  |  |  |  |
| Yes | 1.17(0.63,2.16) | 0.623 | 0.312 | 1.19(0.64,2.20) | 0.589 | 0.271 | 1.27(0.60,2.71) | 0.532 | 0.514 | 1.35(0.63,2.90) | 0.4373 | 0.523 |
| No | **1.76(1.07,2.89)** | **0.027** |  | **1.82(1.11,3.00)** | **0.019** |  | 1.71(0.93,3.14) | 0.083 |  | 1.70(0.92,3.11) | 0.0883 |  |
| Elevated triglycerides(mg/dl) |  |  |  |  |  |  |  |  |  |  |  |  |
| Yes | 1.33(0.70,2.51) | 0.387 | 0.661 | 1.36(0.72,2.59) | 0.344 | 0.652 | 1.28(0.61,2.71) | 0.511 | 0.619 | 1.26(0.60,2.67) | 0.5414 | 0.617 |
| No | 1.58(0.97,2.58) | 0.064 |  | **1.63(1.00,2.66)** | **0.050** |  | 1.70(0.92,3.13) | 0.090 |  | 1.67(0.91,3.08) | 0.0991 |  |
| Reduced HDL-C(mg/dl) |  |  |  |  |  |  |  |  |  |  |  |  |
| Yes | 0.87(0.46,1.64) | 0.665 | 0.031 | 0.90(0.48,1.71) | 0.754 | 0.031 | 0.82(0.40,1.69) | 0.599 | 0.024 | 0.83(0.41,1.71) | 0.6206 | 0.023 |
| No | **2.13(1.28,3.54)** | **0.004** |  | **2.18(1.31,3.63)** | **0.003** |  | **2.93(1.47,5.84)** | **0.002** |  | **2.89(1.45,5.77)** | **0.0026** |  |
| Metabolic syndrome |  |  |  |  |  |  |  |  |  |  |  |  |
| Yes | 1.21(0.66,2.21) | 0.544 | 0.557 | 1.32(0.72,2.43) | 0.375 | 0.560 | 1.38(0.66,2.88) | 0.393 | 0.383 | 1.38(0.66,2.88) | 0.3927 | 0.383 |
| No | 1.51(0.98,2.32) | 0.062 |  | 1.51(0.98,2.32) | 0.063 |  | 1.53(0.90,2.60) | 0.116 |  | 1.53(0.90,2.60) | 0.1156 |  |

Abbreviations: HR = hazard ratio; CI = confidence interval. Bold values indicate the positive results of the study.

† adjusted for age, gender, educational level, marital status, area of residence, and behaviors including smoking, drinking, and physical activity;

‡ adjusted for age, gender, educational level, marital status, area of residence, behaviors including smoking, drinking, and physical activity, along with additional adjustment for self-reported diagnosis of stroke, dyslipidemia, hypertension, diabetes or high blood sugar, BMI, waist circumference, triglycerides, HDL-C, systolic and diastolic blood pressure, fasting plasma glucose and HbA1c levels.
